# Patient-reported outcomes from STARTRK-2: a global phase II basket study of entrectinib for *ROS1* fusion-positive non-small-cell lung cancer and *NTRK* fusion-positive solid tumours

**DOI:** 10.1016/j.esmoop.2021.100113

**Published:** 2021-04-27

**Authors:** L. Paz-Ares, F. Barlesi, S. Siena, M.-J. Ahn, A. Drilon, A. Conley, C. Rolfo, J. Wolf, T. Seto, R. Doebele, A. Kapre, D. Chen, S. McCallum, S. Osborne, G. Demetri

**Affiliations:** 1 Medical Oncology Department, Hospital Universitario 12 de Octubre, CNIO-H12o Lung Cancer Clinical Research Unit, Universidad Complutense & Ciberonc, Madrid, Spain; 2 Medical Oncology Department, Gustave Roussy Cancer Campus, Villejuif, France; 3 Medical Oncology Department, Niguarda Cancer Center, Grande Ospedale Metropolitano Niguarda, Milan, Italy; 4 Department of Oncology and Hemato-Oncology, Università degli Studi di Milano, Milan, Italy; 5 Samsung Medical Center, Sungkyunkwan University School of Medicine, Seoul, Republic of Korea; 6 Department of Medicine, Memorial Sloan Kettering Cancer Center, New York, USA; 7 Weill Cornell Medical College, New York, USA; 8 Department of Sarcoma Medical Oncology, MD Anderson Cancer Center, University of Texas, Houston, USA; 9 Marlene and Stewart Greenebaum Comprehensive Cancer Center, University of Maryland School of Medicine, Baltimore, USA; 10 Department I of Internal Medicine, Center for Integrated Oncology, University Hospital of Cologne, Cologne, Germany; 11 Department of Thoracic Oncology, National Hospital Organization Kyushu Cancer Center, Fukuoka, Japan; 12 Division of Medical Oncology, University of Colorado, Aurora, USA; 13 Department of Patient-Centered Outcomes Research, Genentech, Inc., South San Francisco, USA; 14 Product Development Oncology, Genentech, Inc., South San Francisco, USA; 15 Medication Safety and Risk Management, Genentech, Inc., South San Francisco, USA; 16 PDMA Operations (Biometrics), F. Hoffmann-La Roche Ltd, Basel, Switzerland; 17 Department of Oncologic Pathology, Dana-Farber Cancer Institute and Ludwig Center at Harvard Medical School, Boston, USA

**Keywords:** entrectinib, *NTRK*, patient-reported outcomes, *ROS1*, tyrosine kinase inhibitor

## Abstract

**Background:**

Patient-reported outcomes (PROs) are increasingly relevant endpoints in clinical trials, contributing to our understanding of risk–benefit profiles, in addition to efficacy and safety data. We investigated the impact of entrectinib on patient-reported symptoms, functioning, and health-related quality of life.

**Patients and methods:**

STARTRK-2 is a phase II basket study in patients with locally advanced/metastatic neurotrophic receptor tyrosine kinase 1/2/3 (*NTRK1/2/3*) and ROS proto-oncogene 1 (*ROS1*) fusion-positive solid tumours. PROs (prespecified secondary endpoint) were evaluated using the European Organization for Research and Treatment of Cancer quality-of-life questionnaire (QLQ-C30), lung cancer module (QLQ-LC13), and colorectal cancer module (QLQ-CR29), and the EuroQoL 5-Dimension 3-Level instruments, completed before cycle 1 day 1 and each subsequent 4-week cycle of entrectinib dosing, and the end of treatment. Adverse events and treatment-related symptoms were assessed in the safety analysis (SA)-PRO population. Tumour-related symptoms, functioning, and global health status were assessed in the efficacy analysis (EA)-PRO population. Data cut-offs: 31 October 2018 *NTRK* cohort; 01 May 2019 *ROS1* cohort.

**Results:**

SA-PRO populations comprised patients with *NTRK* fusion-positive solid tumours (*N* = 88) or *ROS1* fusion-positive non-small-cell lung cancer (*N* = 180) who received one or more doses of entrectinib, completed PRO questionnaires on cycle 1 day 1 and answered one or more questions on-study. EA-PRO populations (*N* = 71) and (*N* = 145), respectively, comprised SA-PRO patients with measurable baseline disease. Moderate-to-high baseline global health status scores were maintained in EA-PRO populations during treatment. Role and physical functioning scores were moderate-to-high at baseline, with trends towards clinical improvement during treatment. Both cohorts reported low-to-moderate symptom burden at baseline, which was maintained or trended towards clinically meaningful improvement. Symptoms commonly associated with cancer treatment (e.g. nausea, fatigue) remained stable or improved during treatment. All SA-PRO patients experienced one or more adverse events, most frequently constipation or diarrhoea.

**Conclusions:**

PRO findings were consistent with the favourable safety profile of entrectinib, and further reinforce the positive benefit–risk profile of this treatment, indicating minimal overall treatment burden.

## Highlights

• During treatment, moderate-to-high baseline functioning and health-related quality-of-life scores were improved/maintained.
• Low-to-moderate tumour-related symptom burden remained stable or trended towards clinical improvement, relative to baseline.
• Notably there was a rapid and sustained clinically meaningful improvement in cough in patients with non-small-cell lung cancer.
• Treatment-related symptoms remained stable or improved; common treatment-related symptoms were absent or low severity.
• PRO data reinforce the positive benefit–risk profile of entrectinib and demonstrate a minimal overall treatment burden.

## Introduction

Neurotrophic receptor tyrosine kinase (*NTRK*) and ROS proto-oncogene 1 (*ROS1*) gene fusions are known oncogenic drivers across a range of tumour types,^1^ which result in constitutively active kinase activity.^2^^,^^3^ The European Society for Medical Oncology recommends *NTRK* testing in advanced solid cancers and *ROS1* testing in advanced non-squamous non-small-cell lung cancer (NSCLC).^4^^,^^5^ Entrectinib is an orally available, potent inhibitor of tropomyosin receptor kinase A/B/C, ROS1, and anaplastic lymphoma kinase (ALK).^6^^,^^7^ In an integrated analysis of three clinical trials (ALKA-372-001 [EudraCT, 2012–000148–88], STARTRK-1 [NCT2097810], and STARTRK-2 [NCT02568267]), entrectinib demonstrated clinically meaningful efficacy for locally advanced/metastatic *NTRK* fusion-positive solid tumours [63.5% objective response rate (ORR), *N* = 74] and *ROS1* fusion-positive NSCLC (67.1% ORR, *N* = 161), with a manageable and favourable safety and tolerability profile.^8^^,^^9^

As a complement to traditional outcomes of efficacy and tolerability, patient-reported outcomes (PROs) regarding symptoms and their functional impact on daily activities and health-related quality of life (HRQoL) are gaining increasing recognition as important clinical trial endpoints.^10^ Appropriate consideration of PROs allows for a more comprehensive assessment of an agent's benefit–risk profile by more fully integrating patients' perspectives, and ensuring treatment benefit is not outweighed by unacceptable tolerability and reduced HRQoL.^11^ To provide information on the overall treatment burden associated with entrectinib, we investigated the impact of treatment on patient-reported symptoms, functioning, and HRQoL in the STARTRK-2 study.

PROs were assessed using the core European Organisation for Research and Treatment of Cancer (EORTC) QoL instrument (QLQ-C30),^12^ which is a widely used HRQoL questionnaire that assesses global health status (GHS), important functioning domains, and common cancer-associated symptoms, as well as two additional supplements, which were EORTC QLQ-LC13 (lung cancer module)^13^ and EORTC QLQ-CR29 [colorectal cancer (CRC) module].^14^ The International Consortium for Health Outcomes Measurement has selected EORTC QLQ-C30, QLQ-CR29, and QLQ-LC13 as standard tools for assessing PROs as outcome indicators for patients with cancer, CRC, and lung cancer, respectively.^15^

## Materials and methods

### Patients and study design

The design of STARTRK-2, an open-label phase II basket study, has been reported previously,^8^^,^^9^ and is outlined in more detail in the Supplementary Appendix, available at https://doi.org/10.1016/j.esmoop.2021.100113. Briefly, this study included patients with locally advanced/metastatic solid tumours harbouring an *NTRK1/2/3*, *ROS1*, or *ALK* gene fusion who received oral entrectinib 600 mg once daily in 4-week cycles. Patients with *ALK* fusion-positive solid tumours were enrolled; however, this arm was discontinued and therefore these data are not included in this analysis.

### Safety assessments

Safety was assessed by physical examination, laboratory tests, monitoring of adverse events (AEs), and clinic visits. AEs were coded using the Medical Dictionary for Regulatory Activities (version 14.0 or higher for individual studies; version 21.0 for the integrated safety analysis) and graded using the National Cancer Institute Common Terminology Criteria for Adverse Events (version 4.03).

### PRO assessments

PROs were prespecified secondary endpoints, evaluated in all enrolled patients who completed questionnaires on electronic devices before entrectinib dosing on day 1 of every 4-week cycle starting at cycle 1, and at the end of treatment. These included the EuroQoL Group 5-Dimension 3-Level version (EQ-5D-3L) (assesses health resource utilisation; results not included in this publication) and QLQ-C30, which includes 30 questions and comprises five functional scales, three symptom scales, a GHS/QoL scale, and six single items. The QLQ-LC13 module includes 13 questions assessing lung cancer-specific symptoms, while the QLQ-CR29 module includes 29 questions assessing CRC-specific symptoms (Supplementary Table S1, available at https://doi.org/10.1016/j.esmoop.2021.100113).

### Statistical analysis

The safety analysis (SA) population included all enrolled patients who received one or more doses of entrectinib. The efficacy analysis (EA) population comprised all patients with measurable baseline disease (by investigator) who received one or more doses of entrectinib. The PRO populations comprised all patients in the EA (EA-PRO population) or the SA (SA-PRO population) populations who completed QLQ-C30 on cycle 1 day 1 and answered one or more questions on an on-study time point thereafter. For NSCLC or CRC baskets, the PRO populations included patients who also completed QLQ-LC13 or QLQ-CR29, respectively, on cycle 1 day 1 and answered one or more questions on an on-study time point thereafter, in addition to QLQ-C30. Analyses of QLQ-C30, QLQ-LC13, and QLQ-CR29 scores were conducted in the EA-PRO population to assess common tumour-related symptoms, functioning, and GHS/QoL. Analyses of QLQ-C30 treatment-related symptoms were conducted in the SA-PRO population. The threshold for data evaluation was ≥25% of the SA-PRO or EA-PRO populations remaining enrolled and participating in the PRO questionnaires, in order to optimise the generalisability of the results. PRO data were summarised with descriptive statistics. Select symptom scales (or single items if there is only one item for that scale) were prespecified for analysis based on their relevance to entrectinib treatment and tumour types evaluated.

PROs were scored according to the developers' scoring manual. All scales and single-item measures were linearly transformed to a score range of 0-100. High scores on functional/GHS scales represent a high level of functioning and high HRQoL, respectively. Conversely, high symptom scores represent greater symptomatology severity. PRO scores are also presented here in comparison to EORTC reference data from the general adult population and patients with NSCLC, as appropriate, to define low, moderate, or high categorisation of scores.^16^ For multi-item subscales, if ≤50% of items were missing at a time point, the multi-item score was calculated on the basis of non-missing items. If >50% of items were missing/a single-item measure was missing, the subscale was considered missing. Interpreting scores was based on the subjective significance questionnaire developed by Osoba et al.^17^ which assessed patients' perceptions of changes. Patients who reported ‘a little’ change for better or worse on a particular scale had QLQ-C30 changes ~5-10. Those reporting ‘moderate’ change had changes ~10-20, and ‘very much’ corresponded to a change >20. A ≥10-point change in a score was therefore the threshold for clinical meaningfulness.^17^

### Ethical approval and consent to participate

The trial was conducted in accordance with Good Clinical Practice guidelines and the provisions of the Declaration of Helsinki. All patients provided written informed consent. An independent data and safety monitoring committee reviewed safety data regularly. Protocol approval was obtained from an independent ethics committee at each site.

## Results

### Patients

Enrolment and data cut-offs were 30 April 2018 and 31 October 2018, respectively, for the *NTRK* fusion-positive cohort and 31 October 2018 and 01 May 2019, respectively, for the *ROS1* fusion-positive cohort (STARTRK-2 enrolment ongoing at time of reporting). The SA-PRO population (*N* = 268) comprised 88 patients with locally advanced/metastatic *NTRK* fusion-positive solid tumours and 180 patients with locally advanced/metastatic *ROS1* fusion-positive NSCLC. The EA-PRO population (*N* = 216) comprised 71 patients with locally advanced/metastatic *NTRK* fusion-positive solid tumours and 145 patients with locally advanced/metastatic *ROS1* fusion-positive NSCLC (Supplementary Figure S1, available at https://doi.org/10.1016/j.esmoop.2021.100113). Baseline patient characteristics are summarised in Supplementary Tables S2 and S3, available at https://doi.org/10.1016/j.esmoop.2021.100113. A threshold of ≥25% of enrolled patients responding to the questionnaire determined the number of cycles included in subsequent analyses. Questionnaire completion rates, which represent patients providing data amongst those expected to complete the questionnaire at each time point (patients who had progressed were removed from the denominator), were high at baseline for the *ROS1* SA-PRO and EA-PRO populations (97.8%-97.9%), and remained high until the latest cycle included in analyses (cycle 18: 90.2%-90.7%). For the *NTRK* SA-PRO and EA-PRO populations, baseline completion rates were high (85.7%-100.0%) and decreased slightly by the latest cycle included in analyses (cycle 12/13: 68.8%-80.0%).

Using normative scores of the EORTC from the general adult population as a reference,^16^ patients in the *NTRK* and *ROS1* EA-PRO populations (*N* = 88, *N* = 145, respectively) reported moderate-to-high QLQ-C30 GHS/QoL and functioning at baseline, with slightly higher scores for the *NTRK* EA-PRO population (baseline scores: GHS/QoL 68.6; physical functioning 74.8; role functioning 66.9; cognitive functioning 83.9) versus the *ROS1* EA-PRO population (baseline scores: GHS/QoL 56.0; physical functioning 71.5; role functioning 62.4; cognitive functioning 82.8) (Table 1). Baseline treatment-related symptom scores (QLQ-C30) and tumour-related symptom scores (QLQ-LC13) were lower or comparable with normed scores for patients with NSCLC^16^ (Table 1).

**Table 1:** Mean baseline scores and mean change from baseline in global health status, functioning (based on QLQ-C30), and tumour-related symptom severity (based on QLQ-LC13 and QLQ-CR29), in the *NTRK* EA-PRO population (*N* = 71) and *ROS1* EA-PRO population (*N* = 145), and treatment-related symptom severity (based on QLQ-C30) in the *NTRK* SA-PRO population (*N* = 88) and *ROS1* SA-PRO population (*N* = 180)

QLQ-C30 functioning scales	*NTRK* fusion-positive solid tumours			*ROS1* fusion-positive NSCLC		
	*NTRK* EA-PRO population (*N* = 71)			*ROS1* EA-PRO population (*N* = 145)		
	BL score (*n* = 65)	Change from BL, C13^a^ (*n =* 19)	Change from BL, range (C2–13^a^)	BL score (*n =* 142)	Change from BL, C18^a^ (*n =* 37)	Change from BL, range (C2–18^a^)
GHS/QoL	68.6	+5.3	+5.3 to −4.4	56.0	+4.1	+11.3 to +3.8
Physical functioning	74.8	+6.3	+7.7 to −0.4	71.5	+5.7	+8.8 to +1.6
Role functioning	66.9	+12.3	+16.0 to −0.6	62.4	+3.6	+10.3 to 0.0
Cognitive functioning	83.9	0.00	+0.8 to −7.6	82.8	−7.7	−3.8 to −10.8

QLQ-LC13 tumour-related symptom scales	*NTRK* NSCLC EA-PRO population (*N =* 12)			*ROS1* NSCLC EA-PRO population (*N =* 145)		
	BL score (*n =* 12)	Change from BL, C13^a^ (*n =* 3)	Change from BL, range (C2–13^a^)	BL score (*n =* 140)	Change from BL, C18^a^ (*n =* 36)	Change from BL, range (C2–18^a^)
Coughing	38.9	−11.1	0.0 to −16.7	38.6	−24.1	−13.6 to −24.1
Chest pain	5.6	0.0	+16.7 to −8.3	18.6	−3.7	−2.2 to −12.5
Dyspnoea	26.9	−7.4	+9.3 to −7.4	32.3	−6.2	−3.6 to −10.3
Arm/shoulder pain	27.8	−22.2	−5.6 to −25.9	18.6	−5.6	−2.3 to −11.6
Pain in other parts	33.3	+11.1	+16.7 to −18.5	24.9^b^	−1.9^c^	+2.2 to −9.2
Peripheral neuropathy	13.9	0.0	+22.2 to 0.0	14.1	0.0	+8.7 to 0.0
Dysphagia	2.8	0.0	+12.1 to 0.0	7.1	+5.6	+10.8 to +5.0

QLQ-CR29 tumour-related symptom scales	*NTRK* CRC EA-PRO population (*N =* 7)		
	BL score (*n =* 6)	Change from BL, C6^a^ (*n =* 2)	Change from BL, range (C2–6^a^)
Abdominal pain	27.8	+16.7	+16.7 to −8.3
Bloating	33.3	+50.0	+50.0 to −16.7
Stool frequency	13.9	0.0	+12.5 to −4.2

QLQ-C30 treatment-related symptom scales	*NTRK* SA-PRO population (*N =* 88)			*ROS1* SA-PRO population (*N =* 180)		
	BL score (*n =* 82)	Change from BL, C12^a^ (*n =* 22)	Change from BL, range (C2–12^a^)	BL score (*n =* 176)	Change from BL, C18^a^ (*n =* 49)	Change from BL, range (C2–18^a^)
Fatigue	37.8	−13.6	+0.2 to −13.6	39.0	−11.8	−3.1 to −11.8
Nausea/vomiting	6.9	−1.5	+2.6 to −3.8	10.8	−6.8	−3.2 to −7.5
Insomnia	28.1	−9.1	−4.8 to −14.1	32.0	−15.0	−11.8 to −20.4
Appetite loss	21.1	−18.2	−10.0 to −18.2	28.0	−15.7	−14.3 to −22.2
Constipation	16.3	−6.1	+16.7 to −6.1	16.7	+11.6	+22.2 to +8.3
Diarrhoea	8.9	+13.6	+13.6 to +3.2	7.4	+10.2	+13.4 to +6.1

All scores are mean or mean change from baseline. Scores range from 0 to 100. For functioning and GHS/QoL, higher score = better HRQoL and function (improvement). For a treatment-related or tumour-related symptom scale/item, however, lower score = lower symptom severity (improvement). A ≥10-point change in score is the threshold for clinical meaningfulness.

BL, baseline; C2, cycle 2 day 1; C6, cycle 6 day 1; C13, cycle 13 day 1; C18, cycle 18 day 1; CRC, colorectal cancer; EA, efficacy analysis; GHS, global health status; NSCLC, non-small-cell lung cancer; *NTRK,* neurotrophic receptor tyrosine kinase; PRO, patient-reported outcomes; QoL, quality of life; *ROS1,* ROS proto-oncogene 1; SA, safety analysis.

a Cycle shown represents when ≥25% of the population (either the EA-PRO or SA-PRO) remained in the study.

b *n =* 139.

c *n =* 35.

### PROs in patients with NTRK fusion-positive solid tumours

**Figure 1:**
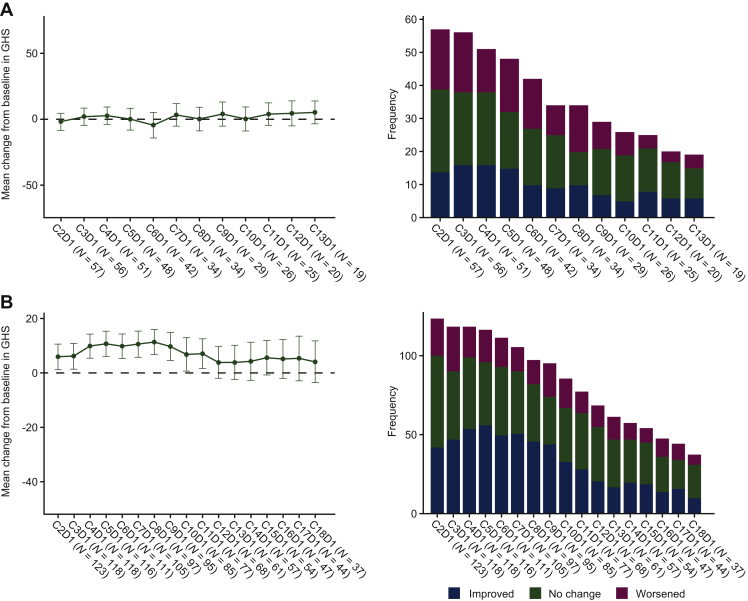
Mean change (with 95% CI) from baseline in global health status (left) and proportion of patients with clinically meaningful change in global health status (right) based on QLQ-C30 in the EA-PRO population with (A) *NTRK* fusion-positive solid tumours (*N =* 71) and (B) *ROS1* fusion-positive NSCLC (*N =* 145). Scores range from 0 to 100. A high score for the GHS/QoL scale represents a high QoL. A ≥10-point change in score is the threshold for clinical meaningfulness. Only visits with three or more patients are included. C, cycle; CI, confidence interval; D, day; EA, efficacy analysis; GHS, global health status; NSCLC, non-small-cell lung cancer; *NTRK*, neurotrophic receptor tyrosine kinase; PRO, patient-reported outcomes; QoL, quality of life; *ROS1*, ROS proto-oncogene 1.

**Figure 2:**
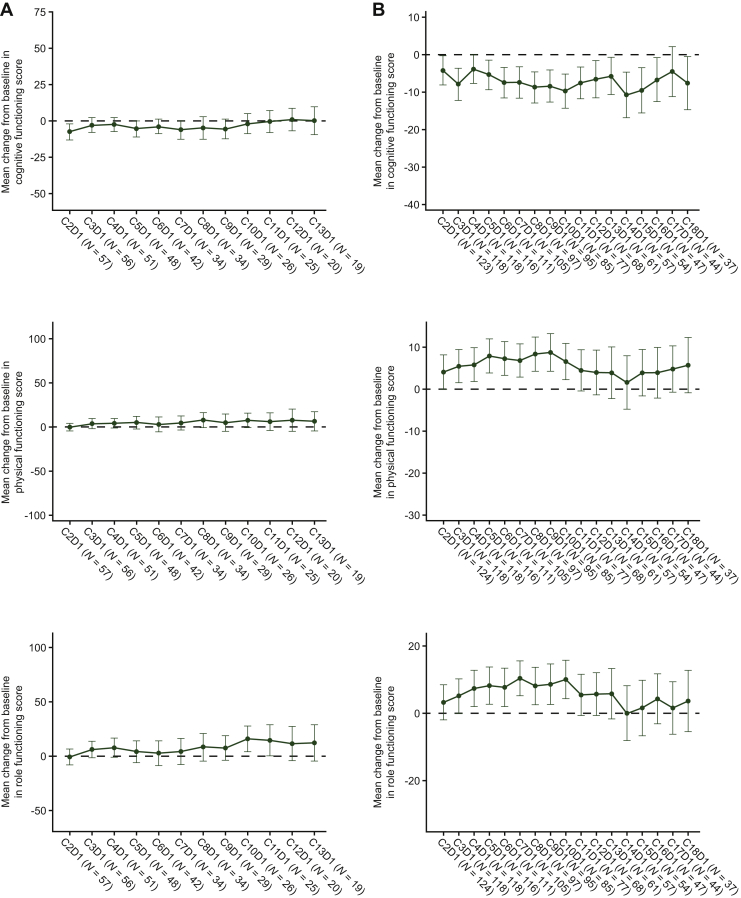
Mean change from baseline in cognitive, physical, and role functioning scores based on QLQ-C30 in the EA-PRO population with (A) *NTRK* fusion-positive solid tumours (*N =* 71) and (B) *ROS1* fusion-positive NSCLC (*N =* 145). Scores range from 0 to 100. A high score for functional scales represents a high/healthy level of functioning. A ≥10-point change in score is the threshold for clinical meaningfulness. Only visits with three or more patients are included. C, cycle; D, day; EA, efficacy analysis; NSCLC, non-small-cell lung cancer; *NTRK*, neurotrophic receptor tyrosine kinase; PRO, patient-reported outcomes; *ROS1*, ROS proto-oncogene 1.

Median treatment duration in the *NTRK* EA population (*N* = 71) was 8.5 months. According to the QLQ-C30, the EA-PRO population maintained a high baseline GHS/QoL [68.6; mean change from baseline range: +5.3 to −4.4 (Table 1; Figure 1A)] as well as a high baseline physical functioning score (74.8; mean change from baseline: +7.7 to −0.4) during treatment (Table 1, Figure 2A). Notably, while there was a moderate baseline role functioning score (66.9), this reached a clinically meaningful improvement by cycle 10 day 1, which was maintained until cycle 13 day 1 [mean change from baseline: +12.3 (Table 1, Figure 2A)]. Cognitive functioning was maintained at or just below the high baseline level of 83.9 [mean change from baseline: +0.8 to −7.6 (Table 1, Figure 2A)].

**Figure 3:**
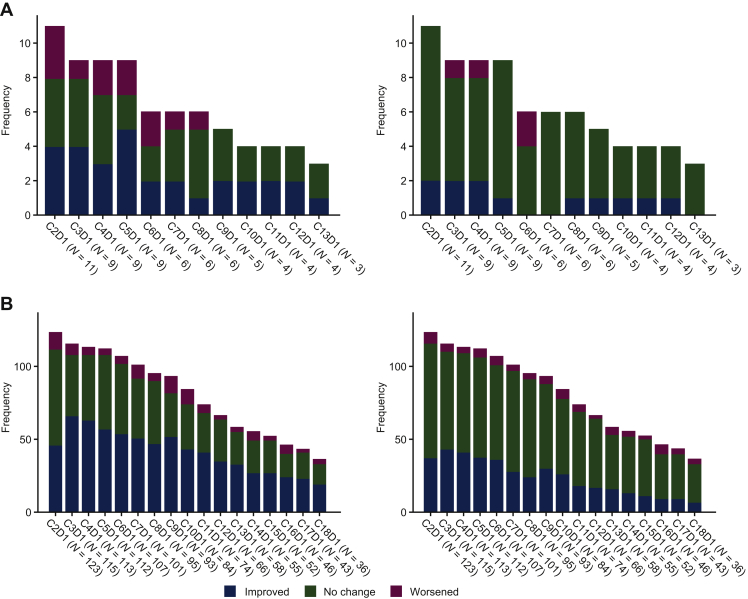
Proportion of patients with clinically meaningful changes from baseline in selected tumour-related symptoms based on QLQ-LC13 in the EA-PRO population with (A) *NTRK* fusion-positive NSCLC [coughing (left), pain in chest (right); *N =* 12] and (B) *ROS1* fusion-positive NSCLC [coughing (left), pain in chest (right); *N =* 145]. Scores range from 0 to 100. A high score for a tumour-related symptom scale/item represents a high level of symptomatology. A ≥10-point change in score is the threshold for clinical meaningfulness. Only visits with three or more patients are included. C, cycle; D, day; EA, efficacy analysis; NSCLC, non-small-cell lung cancer; *NTRK*, neurotrophic receptor tyrosine kinase; PRO, patient-reported outcomes; *ROS1*, ROS proto-oncogene 1.

The *NTRK* NSCLC EA-PRO population (*n* = 12) had a median treatment duration of 7.9 months. Based on the QLQ-LC13, a moderate-to-low baseline tumour-related symptom burden was unchanged or improved throughout treatment (Table 1). There was a low mean baseline score for chest pain (5.6) that remained stable (one exception at cycle 6 day 1: +16.7 mean change from baseline), while dyspnoea remained stable throughout, from a low baseline mean score of 26.9. Cough had a moderate baseline mean score (38.9) with clinically meaningful improvements in symptom severity from cycle 3 day 1 (Figure 3A; mean change from baseline: −14.8), which was maintained at cycle 13 day 1 (mean change from baseline: −11.1). The *NTRK* CRC EA-PRO population (*n* = 6), had median treatment duration of 4.2 months. Based on the QLQ-CR29, CRC-associated symptom severity (*n* = 7, including one patient with neuroendocrine colon cancer) generally remained unchanged or improved over time, in particular bloating, blood/mucus in stool, buttock pain, dyspareunia, dysuria, faecal incontinence, and flatulence (data not shown).

Patients in the *NTRK* SA-PRO population (*N* = 88) reported rapid and durable clinically meaningful improvements in some QLQ-C30 treatment-related symptom scores, where improvements exceeded the clinically meaningful threshold at cycle 12 day 1 for both fatigue and appetite loss. With the exception of diarrhoea, other key treatment-related symptoms, including nausea, insomnia, and constipation, remained stable (Table 1). Based on QLQ-C30 and QLQ-LC13, in the *NTRK* SA-PRO population (*N* = 88) the high proportion of patients reporting treatment-related symptoms ‘not at all’ in the past week remained stable or increased for most symptoms, with no reports of ‘very much’ for any symptom at cycle 12 day 1. The exception was a trend towards decreased reports in the ‘not at all’ category and increased reports in the ‘a little’ category for diarrhoea, constipation, and peripheral neuropathy (Supplementary Table S4, available at https://doi.org/10.1016/j.esmoop.2021.100113).

### PROs in patients with ROS1 fusion-positive NSCLC

Patients in the *ROS1* EA population (*N* = 145) had a median treatment duration of 10.4 months. Based on the QLQ-C30, the EA-PRO population maintained or improved both the moderate baseline GHS/QoL score [56.0; mean change from baseline range: +11.3 to +3.8 (Table 1, Figure 1B)] and moderate baseline role functioning score (62.4; mean change from baseline range: +10.3 to 0.0). A high physical functioning baseline score (71.5) was maintained at most study visits, with a trend towards clinical improvement (Table 1, Figure 2B). A high baseline cognitive functioning score of 82.8 also remained stable throughout, with the exception of cycle 14 [worst mean change score from baseline: −10.8 at cycle 14 day 1 (Table 1, Figure 2B)].

According to the QLQ-LC13, the *ROS1* EA-PRO population (*N* = 145) reported a low-to-moderate tumour-related symptom burden which remained stable or improved throughout. Coughing had a moderate baseline score (38.6), with an immediate clinically meaningful marked improvement (mean change in score from baseline: −13.6 at cycle 2 day 1), sustained throughout [mean change from baseline: −24.1 at cycle 18 day 1 (Table 1, Figure 3B)]. A low baseline chest pain score (18.6) improved or remained stable throughout treatment, including an early clinically meaningful improvement [mean change from baseline: −12.5 at cycle 3 day 1, sustained until cycle 6 day 1: −10.6 (Table 1, Figure 3B)]. Dyspnoea had a moderate baseline score, with a consistent trend towards improvement during treatment, while peripheral neuropathy and dysphagia had low baseline scores, which remained stable with a trend towards worsening in severity (Table 1).

Based on the QLQ-C30, the *ROS1* SA-PRO population (*N* = 180) in general reported a stable or improved low-to-moderate treatment-related symptom burden (Table 1). Fatigue had a moderate baseline score of 39.0, which remained stable with a trend towards improvement. Appetite loss (baseline: 28.0) and insomnia (baseline: 32.0) had immediate marked clinically meaningful improvements (mean changes from baseline: −14.3 and −11.8, respectively, at cycle 2 day 1), which were maintained at cycle 18 day 1 (mean changes from baseline: −15.7 and −15.0, respectively). A low baseline nausea symptom score was maintained during treatment. Constipation (baseline score: 16.7) demonstrated a sustained clinically meaningful worsening over time, while the low baseline diarrhoea score (7.4) remained stable until cycle 9 day 1 before clinically meaningful symptom worsening until cycle 18 day 1 (Table 1). Based on QLQ-C30 and QLQ-LC13, in the *ROS1* SA-PRO population (*N* = 180 and *N* = 145, respectively), the high proportion of patients at baseline reporting treatment-related symptoms ‘not at all’ in the past week generally remained stable, or increased by cycle 18 day 1 (including appetite loss, nausea, vomiting, trouble sleeping, tingling hands and feet, sore mouth). Reports remained stable or decreased for ‘quite a bit’, and ‘very much’ at cycle 18 day 1 for these symptoms. The exceptions were constipation, diarrhoea, and hair loss, which had decreased reports of ‘not at all’ by cycle 18 day 1 (Supplementary Table S4, available at https://doi.org/10.1016/j.esmoop.2021.100113).

### Safety

All patients in the SA-PRO populations experienced at least one AE; the most frequently reported AEs (any grade) associated with QoL impairment were constipation (45.5% of the *NTRK* SA-PRO population and 52.2% of the *ROS1* SA-PRO population), diarrhoea (38.6% and 40.0%, respectively), nausea (25.0% and 31.1%, respectively), and dyspnoea (25.0% and 31.7%, respectively). Treatment-related AEs leading to discontinuations occurred in 4.5% of the *NTRK* SA-PRO population and 5.6% of the *ROS1* SA-PRO population. There were generally no notable differences in the AE profile between the *NTRK* and *ROS1* SA-PRO populations (Table 2).

**Table 2:** Summary of safety in the *NTRK* and *ROS1* SA-PRO population

Patients, *n* (%)	*NTRK* fusion-positive solid tumours (*N =* 88)	*ROS1* fusion-positive NSCLC (*N =* 180)
Median, months (range)	14.7 (0.1^a^-29.7)	16.8 (0.1^a^-37.8)
Patients with at least one		
AE	88 (100.0)	180 (100.0)
Grade ≥3 AE	66 (75.0)	120 (66.7)
Serious AE	46 (52.3)	75 (41.7)
TRAEs ≥grade 3	35 (39.8)	68 (37.8)
TRAEs leading to dose reduction	28 (31.8)	54 (30.0)
TRAEs leading to discontinuation	4 (4.5)	10 (5.6)
Study status at data cut-off		
Ongoing/discontinued	49 (55.7)/39 (44.3)	108 (60.0)/72 (40.0)
Discontinuation due to death	33 (84.6)	50 (69.4)
Discontinuation due to consent withdrawal	5 (12.8)	18 (25.0)
Discontinuation due to loss to follow-up	1 (2.6)	2 (2.8)
Discontinued, other	–	2 (2.8)
AEs associated with QoL impairment (any grade)		
Constipation	40 (45.5)	94 (52.2)
Diarrhoea	34 (38.6)	72 (40.0)
Dyspnoea	22 (25.0)	57 (31.7)
Nausea	22 (25.0)	56 (31.1)
Vomiting	17 (19.3)	41 (22.8)
Decreased appetite	9 (10.2)	23 (12.8)
Dyspnoea exertional	2 (2.3)	3 (1.7)

AE, adverse event; NSCLC, non-small-cell lung cancer; *NTRK,* neurotrophic receptor tyrosine kinase; PRO, patient-reported outcomes; QoL, quality of life; SA, safety analysis; *ROS1,* ROS proto-oncogene 1; TRAE, treatment-related AE.

a Censored.

## Discussion

Here we present PRO data from STARTRK-2, which provide additional evidence to support the association of entrectinib with clinically meaningful benefit in patients with *NTRK* fusion-positive solid tumours or *ROS1* fusion-positive NSCLC.^8^^,^^9^ STARTRK-2 safety data demonstrate entrectinib has a manageable and relatively favourable safety profile. Overall, these PRO data support cumulative safety data, and are consistent with the overall entrectinib benefit–risk assessment. Importantly, patient-reported functioning and HRQoL remained stable or showed a trend towards improvement while on treatment. Patients with *ROS1* fusion-positive NSCLC reported moderate-to-low treatment-related and tumour-related symptom burdens throughout, which remained stable or showed a trend towards improvement in most categories, with clinically meaningful improvements in lung-related symptom severity, in particular coughing, as well as some treatment-related symptoms such as appetite loss and trouble sleeping. There were only a few exceptions where there was a trend towards worsening (peripheral neuropathy and dysphagia) or sustained clinically meaningful worsening (constipation and diarrhoea). Patients with *NTRK* fusion-positive NSCLC or CRC reported moderate-to-low treatment- and tumour-related symptom burdens throughout, with either stable or clinically meaningful improvements in key symptoms. High questionnaire response rates indicate that the data are representative of the overall trial population.

For this analysis, PRO symptoms for evaluation were chosen based on the safety/efficacy profile of entrectinib, which indicated that insomnia, appetite loss, nausea, vomiting, constipation, diarrhoea, peripheral neuropathy, and sore mouth were potentially bothersome. In both SA-PRO populations, reports indicate no notable increase in the severity of these symptoms during treatment. Results of this analysis therefore demonstrate that these symptoms had minimal impact on patients' daily lives and HRQoL, providing further support for the safety and tolerability of entrectinib.

Similar to QLQ-C30 and QLQ-C13 findings for a phase II study of alectinib in patients with *ALK* fusion-positive advanced NSCLC,^18^ entrectinib was associated with a trend towards improvement in a number of lung cancer-related symptoms, such as cough, dyspnoea, and chest pain, a sustained high HRQoL, and PRO data were consistent with previously reported safety data. The alectinib study shows that a different tyrosine kinase inhibitor in a similar patient population also demonstrates the capacity to improve GHS/QoL and functioning, as well as alleviate the tumour-related symptom burden, while contributing a minimal treatment-related symptom burden. The value of assessing PROs to generate a more informed benefit–risk profile has also been demonstrated for other targeted therapies. Crizotinib and lorlatinib study PRO assessments demonstrated maintained or improved GHS, global QoL, physical functioning and symptoms, e.g. fatigue, cough, pain, and dyspnoea, but some worsening of specific treatment-related symptoms.^19^^,^^20^

Safety findings demonstrate low discontinuation rates due to treatment-related AEs. Rates of protocol-governed dose reduction were similar in the *NTRK* and *ROS1* SA-PRO populations (31.8% and 30.0%, respectively). However, dose intensity was maintained throughout, with a median dose intensity of 91.4% (*NTRK* EA population; *N* = 71) and 92.9% (*ROS1* EA population; *N* = 145), indicating that for the majority of patients, dose modifications were generally short term and did not result in a notable decrease in entrectinib treatment received throughout the study.

Strengths of this entrectinib PRO analysis include the use of multiple validated questionnaires and the high completion rates, as well as alignment with entrectinib's clinical trial efficacy and safety profile. However, some caution may be needed when interpreting results due to the small sample sizes. The single-arm STARTRK-2 trial design and lack of blinding could also potentially create bias in self-reporting, based on patients' treatment expectations.

## Conclusions

Entrectinib has a manageable and relatively favourable safety and tolerability profile, which was similar for patients with *NTRK* fusion-positive solid tumours or *ROS1* fusion-positive NSCLC. PRO findings are consistent with this profile, and further reinforce the positive benefit–risk of this treatment. In conclusion, from the patient's perspective, the overall treatment burden is minimal with entrectinib.
