# Supplementary material for: Patient-reported outcomes from STARTRK-2: a global phase II basket study of entrectinib for ROS1 fusion-positive non-small-cell lung cancer and NTRK fusion-positive solid tumours
Source: ESMO Open. 2021 Apr 27;6(3):100113. doi: 10.1016/j.esmoop.2021.100113 (PMC8100628; doi:10.1016/j.esmoop.2021.100113)
Supplement: Supplementary Appendix [file mmc1.docx]

**Supplementary appendix**

**Supplementary methods**

Briefly, this study included patients with histologically or cytologically confirmed locally advanced or metastatic solid tumours harbouring a neurotrophic receptor tyrosine kinase (*NTRK)1/2/3*, ROS proto-oncogene 1 (*ROS1)*, or anaplastic lymphoma kinase *(ALK)* gene fusion (with and without central nervous system [CNS] involvement) and an Eastern Cooperative Oncology Group performance status of 0, 1, or 2. Any previous therapy, except tropomyosin receptor kinase (TRK), ROS1, or ALK inhibitors, was permitted, and patients with *ALK* or *ROS1* fusion-positive non-small cell lung cancer who had previously been treated with crizotinib and had experienced CNS-only progression were permitted to enrol. As a basket trial, patients enrol in one of several single arms and receive entrectinib; there is no comparator arm. All patients received oral entrectinib 600 mg once daily in 4-week cycles, remaining on treatment until documented radiographic progression, as assessed by blinded independent central review (BICR), development of unacceptable toxicity, or withdrawal of consent. The primary endpoint was BICR-assessed overall response rate. Secondary efficacy endpoints include best overall response, duration of response, time to response, progression-free survival (PFS), clinical benefit rate, intracranial tumour response, intracranial PFS, and overall survival. STARTRK-2 is ongoing at >150 sites (including cancer and medical centres, research institutes, hospitals, and universities) in 15 countries.

**Supplementary Table S1. PRO instruments: functional/symptom domains and single items**

| **QLQ-C30** | | |
| --- | --- | --- |
| **Functional domains** | **Symptom domains and single items** | **Additional scale** |
| Physical  Role  Cognitive  Emotional  Social | Fatigue  Pain  Nausea/vomiting  Dyspnoea  Loss of appetite  Insomnia  Constipation  Diarrheal | GHS/QoL |
| **EORTC QLQ-CR29** | | |
| **Symptom domains** | **Single Items** |  |
| Body image  Anxiety  Weight  Sexual interest (men)  Sexual interest (women) | Urinary frequency  Blood and mucus in stool  Stool frequency  Urinary incontinence  Dysuria  Abdominal pain  Buttock pain  Bloating  Dry mouth  Hair loss  Taste  Flatulence  Faecal incontinence  Sore skin  Embarrassment  Stoma care problems  Impotence  Dyspareunia |  |
| **EORTC QLQ-LC13** | | |
| **Symptom domain** | **Single items** |  |
| Dyspnoea | Coughing  Haemoptysis  Sore mouth  Dysphagia  Peripheral neuropathy  Alopecia  Pain in chest  Pain in arm/shoulder  Pain in other parts |  |

EORTC, European Organization for Research and Treatment of Cancer; GHS, global health status; PRO, patient-reported outcomes; QoL, quality of life.

**Supplementary Table S2.** Baseline characteristics of the *NTRK* and *ROS1* SA-PRO populations

| **Characteristic** | ***NTRK* fusion-positive solid tumours (N*=*88)** | ***ROS1* fusion-positive NSCLC (N*=*180)** |
| --- | --- | --- |
| Median age, years (range) | 57 (21–83) | 54 (15–86) |
| Sex, n (%) |  |  |
| Female | 47 (53.4) | 108 (60.0) |
| Male | 41 (46.6) | 72 (40.0) |
| Race, n (%) |  |  |
| Asian | 15 (17.0) | 80 (44.7) |
| Black – African American | 3 (3.4) | 10 (5.6) |
| White | 60 (68.2) | 76 (42.5) |
| Other/unknown |  | 3 (1.7) |
| Not reported | 10 (11.4) | 10 (5.6) |
| ECOG PS, n (%) |  |  |
| 0 | 33 (37.5) | 76 (42.2) |
| 1 | 41 (46.6) | 84 (46.7) |
| 2 | 12 (13.6) | 18 (10.0) |
| 3  4 | 2 (2.3)  0 | 1 (0.6)  1 (0.6) |
| Baseline CNS lesions (INV), n (%)  Measurable  Present  Absent | 8 (9.1)  20 (22.7)  60 (68.2) | 23 (12.8)  51 (28.3)  106 (58.9) |

CNS, central nervous system; ECOG PS, Eastern Cooperative Oncology Group performance status; INV, investigator-assessed; NSCLC, non-small-cell lung cancer; *NTRK*, neurotrophic receptor tyrosine kinase; PRO, patient-reported outcomes; *ROS1*, *ROS* proto-oncogene 1; SA, safety analysis.

**Supplementary Table S3. Baseline characteristics of the *NTRK* and *ROS1* EA-PRO populations**

| **Characteristic** | ***NTRK* fusion-positive solid tumours (N*=*71)** | ***ROS1* fusion-positive NSCLC (N*=*145)** |
| --- | --- | --- |
| Median age, years (range) | 57 (21–83) | 54 (20–86) |
| Sex, n (%) |  |  |
| Female | 37 (52.1) | 93 (64.1) |
| Male | 34 (47.9) | 52 (35.9) |
| Race, n (%) |  |  |
| Asian | 13 (18.3) | 68 (46.9) |
| Black – African American | 2 (2.8) | 7 (4.8) |
| White | 49 (69.0) | 60 (41.4) |
| Other | – | 2 (1.4) |
| Not reported | 6 (8.5) | 8 (5.5) |
| ECOG PS, n (%) |  |  |
| 0 | 30 (42.3) | 61 (42.1) |
| 1 | 31 (43.7) | 69 (47.6) |
| 2 | 10 (14.1) | 15 (10.3) |
| Baseline CNS lesions (INV), n (%)  Measurable  Present  Absent | 1 (1.4)  17 (23.9)  53 (74.6) | 11 (7.6)  40 (27.6)  94 (64.8) |

CNS, central nervous system; EA, efficacy analysis; ECOG PS, Eastern Cooperative Oncology Group performance status; INV, investigator-assessed; NSCLC, non-small cell lung cancer; *NTRK*, neurotrophic receptor tyrosine kinase; PRO, patient-reported outcomes; *ROS1*, *ROS* proto-oncogene 1.

**Supplementary Table S4. Categorical responses of selected treatment-related symptoms over time in the *NTRK* SA-PRO population (N*=*88) and *ROS1* SA-PRO population (N*=*180) according to QLQ-C30 and QLQ-LC13.**

| Categorical response provided by patients with *NTRK* fusion-positive solid tumours | | | | | | | | |
| --- | --- | --- | --- | --- | --- | --- | --- | --- |
| **Symptom, n (%)** | **“Not at all”** | | **“A little”** | | **“Quite a bit”** | | **“Very much”** | |
|  | **BL** | **C12** | **BL** | **C12** | **BL** | **C12** | **BL** | **C12** |
| Diarrhoea* | 65 (79.3) | 8 (36.4) | 13 (15.9) | 12 (54.5) | 3 (3.7) | 2 (9.1) | 1 (1.2) | 0 |
| Nausea* | 65 (79.3) | 19 (86.4) | 12 (14.6) | 1 (4.5) | 4 (4.9) | 2 (9.1) | 1 (1.2) | 0 |
| Vomiting* | 73 (89.0) | 18 (81.8) | 8 (9.8) | 4 (18.2) | 0 | 0 | 1 (1.2) | 0 |
| Appetite loss* | 46 (56.1) | 18 (81.8) | 24 (29.3) | 4 (18.2) | 8 (9.8) | 0 | 4 (4.9) | 0 |
| Constipation* | 52 (63.4) | 11 (50.0) | 23 (28.0) | 10 (45.5) | 4 (4.9) | 1 (4.5) | 3 (3.7) | 0 |
| Trouble sleeping* | 34 (41.5) | 18 (81.8) | 31 (37.8) | 2 (9.1) | 13 (15.9) | 2 (9.1) | 4 (4.9) | 0 |
| Hair loss^†^ | 9 (69.2) | 3 (75.0) | 2 (15.4) | 1 (25.0) | 1 (7.7) | 0 | 1 (7.7) | 0 |
| Tingling hands / feet^†^ | 10 (76.9) | 2 (50.0) | 2 (15.4) | 2 (50.0) | 0 | 0 | 1 (7.7) | 0 |
| Sore mouth^†^ | 13 (100.0) | 4 (100.0) | 0 | 0 | 0 | 0 | 0 | 0 |
| **Categorical response provided by patients with *ROS1* fusion-positive NSCLC** | | | | | | | | |
|  | **BL** | **C18** | **BL** | **C18** | **BL** | **C18** | **BL** | **C18** |
| Diarrhoea* | 140 (79.5) | 31 (63.3) | 34 (19.3) | 16 (32.7) | 1 (0.6) | 1 (2.0) | 1 (0.6) | 1 (2.0) |
| Nausea* | 113 (64.2) | 41 (83.7) | 51 (29.0) | 6 (12.2) | 11 (6.3) | 2 (4.1) | 1 (0.6) | 0 |
| Vomiting* | 146 (83.0) | 48 (98.0) | 25 (14.2) | 1 (2.0) | 2 (1.1) | 0 | 3 (1.7) | 0 |
| Appetite loss* | 80 (45.5) | 38 (77.6) | 59 (33.5) | 11 (22.4) | 22 (12.5) | 0 | 15 (8.5) | 0 |
| Constipation* | 109 (61.9) | 21 (42.9) | 51 (29.0) | 18 (36.7) | 11 (6.3) | 9 (18.4) | 5 (2.8) | 1 (2.0) |
| Trouble sleeping* | 56 (31.8) | 36 (73.5) | 81 (46.0) | 12 (24.5) | 29 (16.5) | 1 (2.0) | 10 (5.7) | 0 |
| Hair loss^‡^ | 110 (78.6) | 31 (83.8) | 22 (15.7) | 5 (13.5) | 6 (4.3) | 1 (2.7) | 2 (1.4) | 0 |
| Tingling hands / feet^‡^ | 97 (69.3) | 22 (59.5) | 31 (22.1) | 15 (40.5) | 8 (5.7) | 0 | 4 (2.9) | 0 |
| Sore mouth^‡^ | 120 (85.7) | 31 (83.8) | 17 (12.1) | 6 (16.2) | 3 (2.1) | 0 | 0 | 0 |

*QLQ-C30, ^†^QLQ-LC13 (*NTRK* NSCLC SA-PRO population [N*=*13]). ^‡^QLQ-LC13 (*ROS1* SA-PRO population [N*=*145]; 35 patients were excluded from this analysis based on enrolment in a sub-study in Japan, prior treatment with crizotinib (CNS progression only), or being non-evaluable). BL, baseline; C18, Cycle 18 Day 1; C12, Cycle 12 Day 1; CNS, central nervous system; *NTRK,* neurotrophic receptor tyrosine kinase; PRO, patient-reported outcomes; *ROS1,* ROS protooncogene 1 fusion positive; SA, safety analysis.

**Supplementary Figure S1. Schematic of the SA-PRO and EA-PRO populations**


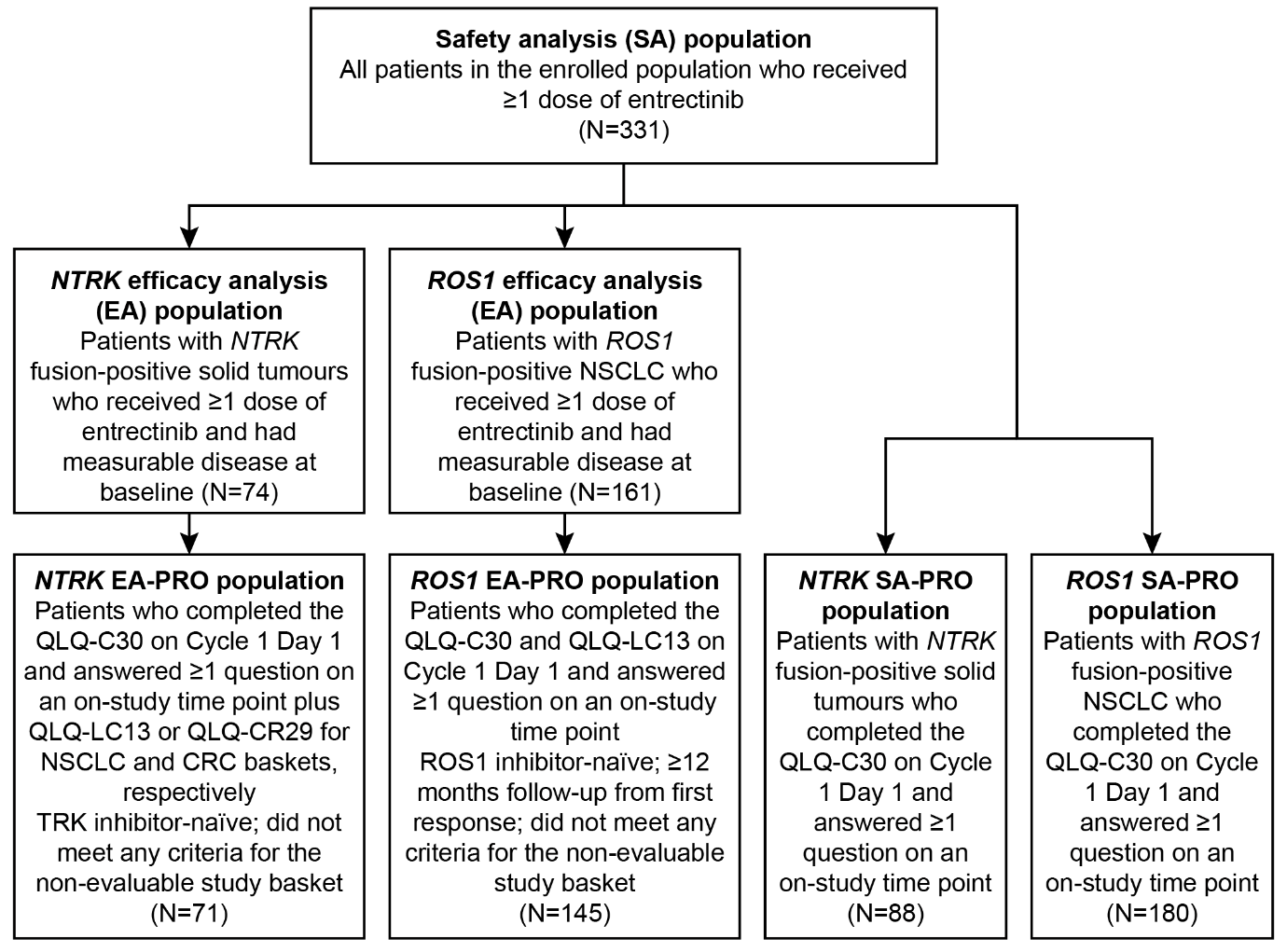


NSCLC, non-small cell lung cancer; *NTRK*, neurotrophic receptor tyrosine kinase; PRO, patient-reported outcomes; *ROS1*, ROS proto-oncogene 1; TRK, tropomyosin receptor kinase.
